# Supplementary material for: Who accepts nudges? nudge acceptability from a self-regulation perspective
Source: PLoS One. 2021 Dec 3;16(12):e0260531. doi: 10.1371/journal.pone.0260531 (PMC8641879; doi:10.1371/journal.pone.0260531)
Supplement: S1 File — (DOCX) [file pone.0260531.s001.docx]

**SUPPLEMENTARY ONLINE MATERIALS**

***Additional models for the subsample of employed participants***

*Table 1.* Linear regression models predicting acceptability, intrusiveness, perceived effectiveness, and goal alignment for the three different types of nudges.

|  | Acceptability | Intrusiveness | Effectiveness | Alignment |
| --- | --- | --- | --- | --- |
|  | *β (SE)* | *β (SE)* | *β (SE)* | *β (SE)* |
| **Default** |  |  |  |  |
| Self-control | .06 (.07) | -.01 (.08) | .07 (.08) | .11 (.07) |
| Proactive Coping | -.01 (.07) | -.02 (.07) | .04 (.08) | -.06 (.07) |
| Self-efficacy | .11 (.08) | .01 (.09) | .06 (.09) | .06 (.08) |
| Perceived Control | -.20 (.07) * | .15 (.07) * | -.06 (.07) | -.11 (.06) |
| Perceived Difficulty | -.03 (.08) | .08 (.08) | -.04 (.08) | -.11 (.07) |
| Autonomous Motivation | .30 (.07) *** | -.22 (.08) ** | .10 (.08) | .35 (.07) *** |
| Controlled Motivation | .01 (.07) | -.02 (.07) | .02 (.07) | .03 (.07) |
|  |  |  |  |  |
| **Portion Size** |  |  |  |  |
| Self-control | .22 (.08) ** | -.17 (.08) * | .19 (.08) * | .21 (.07) ** |
| Proactive Coping | .02 (.07) | -.00 (.08) | -.01 (.08) | -.02 (.07) |
| Self-efficacy | -.20 (.09) * | .12 (.09) | -.07 (.09) | -.22 (.08) * |
| Perceived Control | .04 (.07) | -.01 (.07) | .04 (.07) | .03 (.07) |
| Perceived Difficulty | .05 (.08) | -.00 (.08) | .05 (.08) | .03 (.08) |
| Autonomous Motivation | .25 (.08) ** | -.09 (.08) | .13 (.08) | .30 (.08) *** |
| Controlled Motivation | .08 (.07) | -.08 (.07) | .04 (.07) | .15 (.07) * |
|  |  |  |  |  |
| **Rearrangement** |  |  |  |  |
| Self-control | -.01 (.07) | -.01 (.08) | -.04 (.08) | .04 (.07) |
| Proactive Coping | .13 (.07) | .08 (.07) | .13 (.07) | .01 (.06) |
| Self-efficacy | .05 (.08) | .14 (.09) | .02 (.09) | .06 (.08) |
| Perceived Control | -.07 (.06) | .02 (.07) | .08 (.07) | -.02 (.06) |
| Perceived Difficulty | .08 (.07) | .13 (.08) | -.04 (.08) | .10 (.07) |
| Autonomous Motivation | .50 (.07) *** | -.36 (.08) *** | .13 (.08) | .48 (.07) *** |
| Controlled Motivation | .00 (.06) | .09 (.07) | .10 (.07) | .14 (.06) * |

*Note.* Model fit for Acceptability: *R^2^_adj_* = .12 *** (Default); *R^2^_adj_* = .08 *** (Portion size); *R^2^_adj_* = .26 *** (Rearrangement). Model fit for Intrusiveness: *R^2^_adj_* = .04 * (Default); *R^2^_adj_* = .01 (Portion size); *R^2^_adj_* = .08 *** (Rearrangement). Model fit for Effectiveness: *R^2^_adj_* = .01 (Default); *R^2^_adj_* = .02 (Portion size); *R^2^_adj_* = .05 ** (Rearrangement). Model fit for Alignment: *R^2^_adj_* = .12 *** (Default); *R^2^_adj_* = .10 *** (Portion size); *R^2^_adj_* = .29 *** (Rearrangement).
